# *Mycoplasma agalactiae* Induces Cytopathic Effects in Infected Cells Cultured *In Vitro*

**DOI:** 10.1371/journal.pone.0163603

**Published:** 2016-09-23

**Authors:** Shrilakshmi Hegde, Shivanand Manjunath Hegde, Renate Rosengarten, Rohini Chopra-Dewasthaly

**Affiliations:** Division of Clinical Microbiology and Infection Biology, Institute of Bacteriology, Mycology and Hygiene, Department of Pathobiology, University of Veterinary Medicine Vienna, Vienna, Austria; CEA, FRANCE

## Abstract

*Mycoplasma agalactiae* is the etiological agent of the contagious agalactia syndrome in sheep and goats and causes significant economic losses worldwide. Yet the mechanism of pathogenesis is largely unknown. Even whole-genome sequence analysis of its pathogenic type strain did not lead to any conclusions regarding its virulence or pathogenicity factors. Although inflammation and tissue destruction at the local site of *M*. *agalactiae* infection are largely considered as effects of the host immune response, the direct effect of the agent on host cells is not completely understood. The aim of this study was to investigate the effect of *M*. *agalactiae* infection on the quality and viability of host cells *in vitro*. Changes in cell morphology including cell elongation, cytoplasm shrinkage and membrane blebbing were observed in infected HeLa cells. Chromatin condensation and increased caspase-3 cleavage in infected HeLa cells 48 h after infection suggests an apoptosis-like phenomenon in *M*. *agalactiae*-infected cells. In compliance with these results, decreased viability and cell lysis of *M*. *agalactiae*-infected HeLa cells was also observed. Measurement of the amount of LDH released after *M*. *agalactiae* infection revealed a time- and dose-dependent increase in HeLa cell lysis. A significant decrease in LDH released after gentamicin treatment of infected cells confirmed the major role of cytadherent *M*. *agalactiae* in inducing host cell lysis. This is the first study illustrating *M*. *agalactiae’s* induction of cytopathic effects in infected HeLa cells. Further detailed investigation of infected host tissue for apoptotic markers might demonstrate the association between *M*. *agalactiae*-induced host cell lysis and the tissue destruction observed during *M*. *agalactiae* natural infection.

## Introduction

Mycoplasmas are the simplest and smallest self-replicating bacteria with rather complex and sophisticated pathogenic attributes [1, 2]. Due to regressive evolution, they have lost their cell wall and many metabolic pathways and hence largely depend on their host for many metabolic precursors [3]. As successful pathogens, mycoplasmas have evolved many complex molecular mechanisms, including antigenic variation, mimicking host antigens and immune modulation [4, 5]. Although some progress has been made in the last decade, detailed studies of these features have largely suffered due to their fastidious growth and recalcitrance to genetic manipulations [5, 6].

*Mycoplasma agalactiae* is one of the most successful bacterial pathogens of small ruminants and the main etiological agent of the contagious agalactia syndrome. The latter is mainly characterized by mastitis, arthritis and keratoconjunctivitis, but septicaemia, pneumonia and reproductive disorders have also been reported [7, 8]. Clinical signs in the eyes starting with conjunctivitis can lead to parenchymatous keratitis with corneal revascularisation, which in turn could eventually cause blindness in severe infections [7]. In cases of chronic arthritis, ankyloses have also been reported [7]. Recent studies have revealed that *M*. *agalactiae* is capable of invading host cells and can spread to distant body sites both in naturally and experimentally infected animals [9, 10]. Also *M*. *agalactiae*-specific signals were observed inside epithelial and macrophage cells in tissue samples collected after experimental sheep infection [10]. Antibiotics tend to reduce clinical signs but promote the carrier state. Under stress conditions, these carriers mediate disease outbreaks causing significant economic loss [11, 12].

Mycoplasmas lack typical bacterial virulence factors like toxins, and hence interaction with the host cells is crucial for colonisation and pathogenicity [13]. Mycoplasma attachment to host cells leading to cytopathic effects, such as epithelial damage and ion channel blockage, has been reported in some mycoplasmas due to interference with host membrane receptors [14, 15]. Few other reports have described the release of noxious enzymes like nucleases or cytotoxic metabolites like H_2_O_2_ and peroxides by mycoplasmas to optimize their own growth and metabolism inside host cells for survival [16–18]. Other features of mycoplasmas damaging host cell integrity include competition for precursors, modulating the host immune system and cytolytic enzyme-mediated fusion of mycoplasma cell components with host cells [5, 19, 20]. A few recent studies revealed that some mycoplasmas can cause damage to infected host cells by inducing processes like apoptosis and necrosis [21–23]. However, it has also been shown that some mycoplasma infections actually prevent apoptosis [24], and that the type of anti- or pro-apoptotic effect of mycoplasmas on host cells is species-dependent [21].

The current study investigates for the first time the effect of *M*. *agalactiae* infection on host cell viability at the cellular level using the HeLa cell infection model. Results indicate that *M*. *agalactiae* infection might induce irreversible damage to host cells, and it is likely that this cell damage plays an important role in pathogenesis during *M*. *agalactiae* infection of its natural host.

## Materials and Methods

### Mycoplasma growth conditions

*M*. *agalactiae* type strain PG2 was grown in SP4 medium as described before [25] for 48 h and diluted serially in minimal essential medium (MEM) with 10% heat-inactivated fetal bovine serum (FBS) (Life Technologies) prior to infection of cultured HeLa cells. Late logarithmic phase *M*. *agalactiae* culture was used for infection as this was observed to be the most optimal during our previous studies [10]. The number of colony forming units (CFU) of mycoplasmas at the time of infection was determined by plating serial dilutions on SP4 agar plates containing 1% noble agar (Difco) and counting colonies under a BMS 74955 stereomicroscope after 4–5 days of incubation at 37°C.

### Cell culture and infection

HeLa-229 cells (ATCC CCL-2.1) were purchased from the American Type Culture Collection (ATCC) and were grown in MEM with 10% heat- inactivated FBS and supplemented with non-essential amino acids (all purchased from Life Technologies). Cells were grown at 37°C with 5% CO_2_ and 98% humidity. For fluorescence staining, 1 x 10^4^ HeLa cells were grown on 8-well LabTech chamber slides (Nunc International) and to study caspase-3 cleavage, 2.5 x 10^4^ HeLa cells were seeded in 24-well plates (CELLSTAR^®^, Greiner Bio-One) and grown for 48 h to reach an approximate confluency of 75% before infection with *M*. *agalactiae* to achieve an approximate MOI of 500. As positive control 1 μM staurosporine (Cell Signalling) was used and incubated with HeLa cells for 3 h to induce apoptosis.

### Viable cell counting of *M*. *agalactiae*-infected HeLa cells

HeLa cells (5 x 10^4^) were seeded in 6-well plates (CELLSTAR^®^, Greiner Bio-One) 48 h before infection with *M*. *agalactiae* with an approximate MOI of 200–500. After 0, 24, 48 and 72 h of infection, HeLa cells were trypsinized (Trypsin and PBS were purchased from PAA Laboratories), and the viable cell count was calculated by Trypan blue exclusion staining. As a control, uninfected HeLa cells were cultured in parallel wells, and viable cell counts were calculated as before. The percentage viability difference was calculated as percentage ratio of viable cells in *M*. *agalactiae*-infected wells to the total viable cell counts in parallel uninfected control wells.

### DNA-fluorescence staining

After 48 h of infection with *M*. *agalactiae*, HeLa cells were washed with PBS to remove any serum content, and the cells were permeabilized by incubation with 70% alcohol for 1 min. Cell nuclei were stained with DAPI (Invitrogen, Life Technologies) and mounted using Mowiol solution (Sigma). As controls, uninfected and staurosporine-treated HeLa cells were also stained with DAPI as above. Images were taken using an OLYMPUS AX 70 epi-fluorescence microscope (x100 magnification), and images were visualised using Soft Imaging System Cell magnification (Olympus).

### Caspase-3 cleavage and immunoblotting

After 24 h and 48 h, uninfected and infected HeLa cells were collected after trypsinization and resuspended in REPA buffer (50 mM Tris, 150 mM Nacl, 0.1% SDS, 0.5% Sodium Deoxycholate, 1% Triton X100, 1 mM PMSF) containing 1x protease inhibitor cocktail (Sigma) to lyse the eukaryotic cells. The lysate was then loaded onto SDS-PAGE gels and transferred to a PVDF membrane (Millipore) through Western blotting. Blots were blocked using 5% non-fat milk (Carl Roth) and probed with rabbit anti-cleaved caspase 3 antibody (1:1000) (Cell Signalling) or rabbit anti-vinculin antibody (1:1000) (Santa Cruz Biotech). After incubating with horseradish peroxidase-conjugated rabbit IgG secondary antibody (Abcam), blots were processed using the enhanced chemiluminescence (ECL) Western blotting detection system (Millipore). Images were taken using Gel Doc™ XR+ System (Bio-Rad). Bands were quantified using ImageJ software (National Institutes of Health, USA) and normalised against loading control (vinculin). Mean optical density values were plotted using GraphPad Prism 5 (Graphpad Software).

### Quantification of LDH in cell supernatants

HeLa cell lysis in the presence of *M*. *agalactiae* was measured in terms of release of cytosolic LDH into culture supernatants. LDH was measured using CytoTox 96™ Nonradioactive Cytotoxicity Assay (Promega) according to the instructions of the manufacturer. Briefly, about 2 x 10^3^ HeLa cells were infected with *M*. *agalactiae*, with an approximate MOI of 25, 250 and 500, and the LDH release was measured at 24, 36 and 48 h p.i. Spontaneous release of LDH was obtained with untreated HeLa cells, and maximum release of LDH was measured with lysed uninfected cells by adding 1x lysis buffer supplied by the manufacturer. In parallel, the same infection was carried out except that 400 μg/mL gentamicin was added to all wells 21 h p.i. and incubated at 37°C to kill the extracellular mycoplasmas [10]. The OD was measured at each time point at a wavelength of 590 nm. Percentage cell lysis was calculated by the formula [(Release of LDH from infected cells–(minus) Spontaneous LDH release) / (Maximum LDH release–(minus) Spontaneous LDH release)] x 100.

### Statistical analysis

All experiments were performed at least three times. Viable cell count analysis of HeLa cells was done in duplicates, whereas LDH quantification assays were performed in triplicates. The statistical significance (*p*-values) of the results was calculated based on the paired Student’s *t* test using GraphPad Prism 5 (Graphpad Software). Differences with *p<*0.05 were considered significant.

## Results and Discussion

### *M*. *agalactiae* infection results in slow growth and decreased viability of HeLa cells

**Fig 1:**
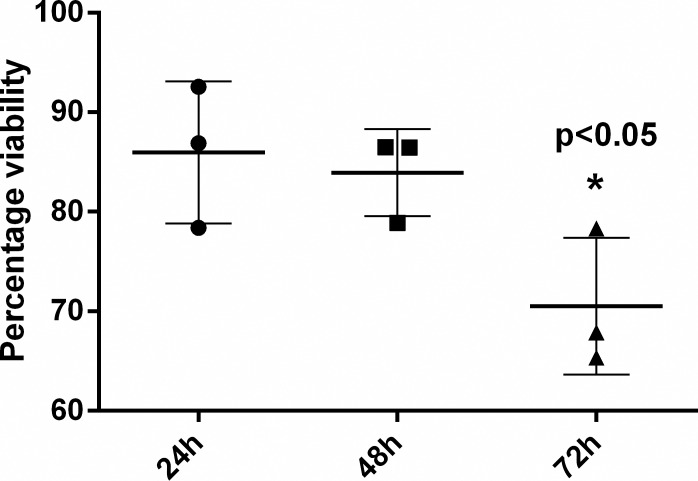
Percentage Viabilty of *M*. *agalactiae*-infected HeLa cells. Viable HeLa cells were measured using Trypan blue staining at 24, 48 and 72 h p.i. Mean values ± SD from three independent experiments performed in duplicates are depicted.

In this study, the effect of *M*. *agalactiae* infection on host cell viability was investigated for the first time. Viable cell counts were compared for HeLa cells in presence and absence of *M*. *agalactiae* using Trypan blue exclusion staining at 0, 24, 48 and 72 h p.i. Compared to the uninfected controls, *M*. *agalactiae*-infected HeLa cells revealed lower viable cell counts at all observed time points, and at 72 h p.i. the number of viable cells was significantly reduced (*p*<0.05). The percentage viability difference between *M*. *agalactiae*-infected cells and uninfected controls became more prominent with increasing time, that is, 85.9 ± 7.1% at 24 h, 83.9 ± 4.3% at 48 h and 70.5 ± 6.8% at 72 h p.i. respectively (Fig 1). A similar effect was also observed by others in similar mycoplasma infection studies involving *M*. *bovis*, *M*. *hyorhinis* and *M*. *synoviae* [22, 23, 26]. As at 72 h p.i. HeLa cells were showing a significant reduction in cell viability, we anticipated that they might be undergoing apoptosis at this time, which was later confirmed by the results presented in the next sections.

### *M*. *agalactiae*-infected HeLa cells exhibit apoptotic phenotype

**Fig 2:**
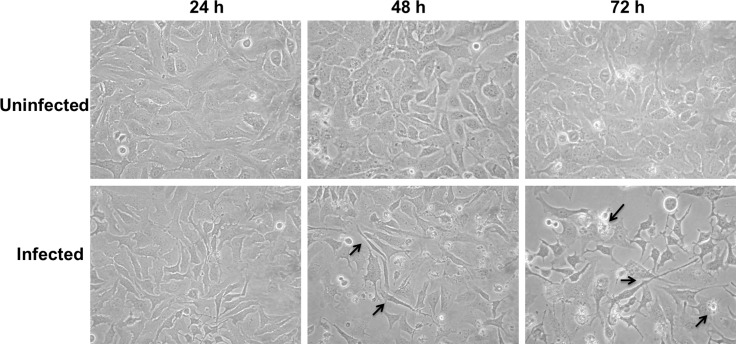
Phase contrast microscopic images showing the morphological changes in *M*. *agalactiae*-infected cells. Images were taken at 24, 48 and 72 h p.i. at 10x magnification. Pointed arrows show the profound cell elongation and membrane blebbing in infected HeLa cells.

**Fig 3:**
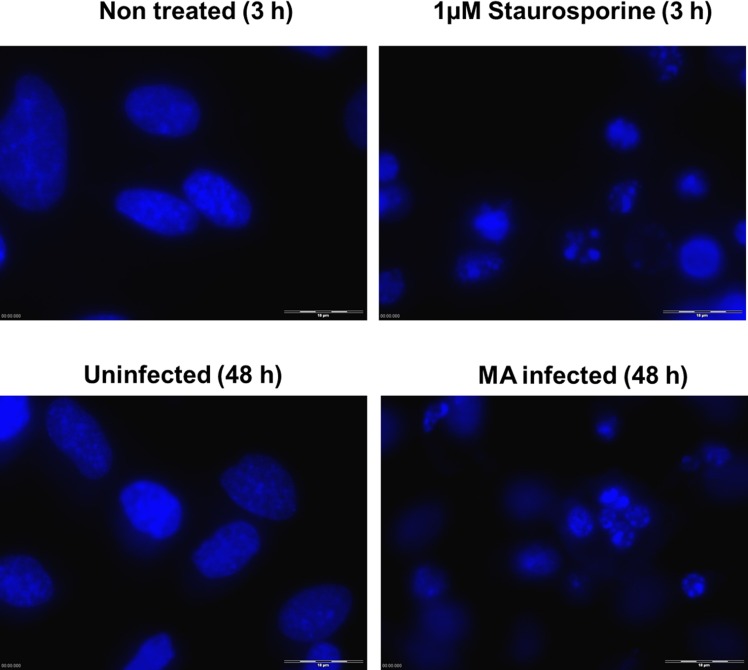
DNA fluorescence staining showing chromatin condensation in *M*. *agalactiae*-infected HeLa cells 48 h p.i. Cells were permeabilized and stained with DAPI, and images were taken using a fluorescence microscope. As a positive control, HeLa cells were treated with 1 μM staurosporine for 3 h and stained with DAPI. Bars, 10 μm.

**Fig 4:**
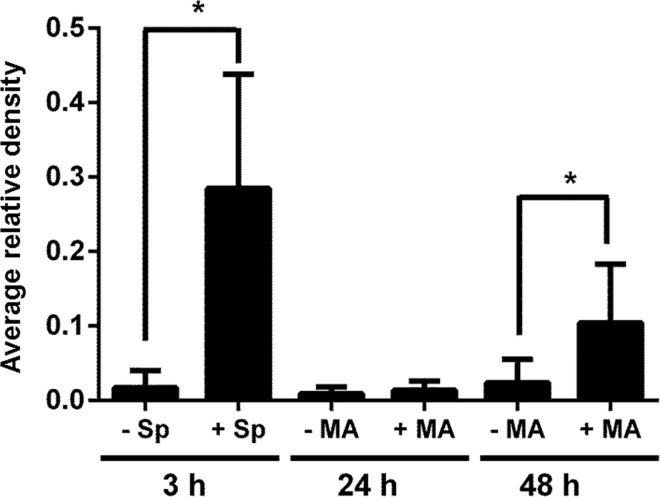
Caspase-3 cleavage in *M*. *agalactiae*-infected HeLa cells. The graph represents the average relative density of cleaved caspase-3 from the immunoblots of *M*. *agalactiae*-infected (+ MA) and uninfected (- MA) HeLa cells at 24 and 48 h. As a positive control, HeLa cells were treated with 1 μM staurosporine (Sp) for 3 h. Experiments were performed at least three times, and average relative density values ± standard deviation are represented here. ‘*’ indicates *p* < 0.05.

Morphological changes in *M*. *agalactiae*-infected HeLa cells were observed using phase contrast microscopy. Cytoplasm shrinkage and membrane blebbing were detected after 48 h of *M*. *agalactiae* infection, and this became even more prominent at 72 h p.i. (Fig 2). Uninfected negative controls did not show any such morphological changes. At 30 h p.i., DAPI-fluorescence staining revealed crescent shaped cell nuclei as an initial characteristic of chromatin condensation (S1 Fig), which was fully established and clearly visible at 48 h p.i. in the DAPI-stained cell nuclei of *M*. *agalactiae*-infected HeLa cells (Fig 3). HeLa cells treated with staurosporine (1 μM) for 3 h served as positive controls and exhibited clear chromatin condensation (Fig 3), whereas uninfected HeLa cells did not show any chromatin abnormality. To further confirm the induction of an apoptosis-like phenomenon, caspase-3 cleavage was checked in *M*. *agalactiae*-infected HeLa cells. There was no caspase-3 cleavage observed 24 h after infection. However, at 48 h p.i. caspase-3 was cleaved and the p17 and p19 fragments were visible using an anti-cleaved caspase-3 antibody (S2 Fig). Quantitative analysis of the caspase-3 cleavage is depicted in Fig 4, where a significant increase in cleavage (*p*<0.05) is visible in *M*. *agalactiae*-infected cells at 48 h p.i. compared to the uninfected cells. This substantiates the ability of *M*. *agalactiae* to induce an apoptosis-like phenomenon in infected HeLa cells.

Both intrinsic and extrinsic signal-mediated apoptosis pathways have been reported in different mycoplasma infections [26, 27]. *M*. *synoviae* and *M*. *hyopneumoniae* use both pathways while inducing apoptosis in host cells [22, 28]. In *M*. *bovis* infection, both an apoptotic and an anti-apoptotic phenomenon have been described using different types of host cells [23, 29]. These reports suggest that mycoplasma- induced cell death is more likely a complex, host micro-environment dependent process. In this study, we have observed caspase-3 cleavage in *M*. *agalactiae*-infected HeLa cells, which is a down-stream step in both intrinsic and extrinsic pathways. Detailed gene expression studies would be helpful in elucidating the role of the exact set of genes involved in *M*. *agalactiae*-induced apoptosis.

### *M*. *agalactiae* induces cell lysis in Infected HeLa cells

**Fig 5:**
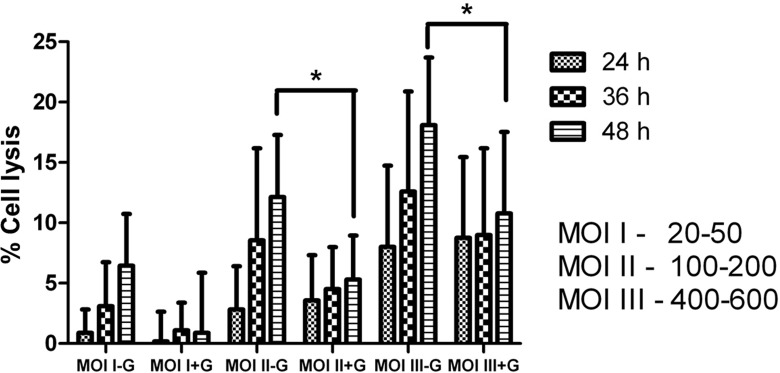
Percentage of cell lysis in *M*. *agalactiae*-infected HeLa cells. Both time- and dose- dependent increase (*p*<0.05) was observed at 36 and 48 h p.i. with all tested MOIs compared to uninfected cells in absence of gentamicin. A significant decrease in cell lysis was observed in gentamicin-treated cells (+G) compared to gentamicin-untreated cells (-G) at 48 h p.i. (‘*’ indicates *p*<0.05).

Cytotoxicity and the ability of a pathogen to induce host cell lysis are often correlated with its pathogenicity. Although cytadhesion is the primary requisite for most mycoplasma infections, local cytotoxic events and tissue damage support disease progression, possibly by providing the necessary precursors for growth and survival of the pathogen [29]. In a previous study, a less virulent PG1 strain of *M*. *mycoides* was found to be deficient in inducing cytotoxicity, thus suggesting its crucial role in virulence of this pathogen [18]. However, the cell lysis and cytotoxicity potential of *M*. *agalactiae* has not been studied so far. Hence, for the first time, we investigated *M*. *agalactiae*-induced cell lysis by quantifying the amount of LDH released in the cell culture supernatants after 24, 36 and 48 h of *M*. *agalactiae* infection (described under Materials and Methods). Time and MOI-dependent increase in the amount of released LDH was observed in the supernatants of infected cell cultures (Fig 5). A significant increase in the amount of released LDH was observed 48 h p.i. at higher MOIs (p<0.05). Killing extracellular mycoplasmas at 24 h p.i. using gentamicin treatment resulted in a significant decrease in the amount of LDH released in the cell culture supernatants at 36 and 48 h p.i. (Fig 5). This suggests that adherent extracellular mycoplasmas play an important role in host cell lysis.

We have demonstrated in an earlier study the ability of *M*. *agalactiae* to invade and exit host cells in a viable state [10], however, the exact mechanisms involved still need to be understood. In this study, the effect of mycoplasma infection on HeLa cell lysis was investigated, and the absence of adherent mycoplasmas was found to result in a significant reduction in HeLa cell lysis. This suggests that adherent extracellular *M*. *agalactiae* could also be capable of damaging host cells during infection. H_2_O_2_ production and nuclease activity, which have been reported in some strains of *M*. *agalactiae* [17, 30], could be the potential factors involved in causing host cell destruction and need to be thoroughly evaluated by detailed studies. However, the involvement of additional mechanisms such as adherence-mediated alterations cannot be ruled out.

## Conclusion

Altogether, this is the first study to show the effect of *M*. *agalactiae* on the viability of cells cultured *in vitro* using HeLa cells as infection model. The decreased cell growth in initial stages of infection may be due to the reduced availability of precursors and nutrients in the presence of *M*. *agalactiae*. But at later stages, along with the decreased HeLa cell counts, chromosome condensation, membrane blebbing and host cell lysis were also observed suggesting the involvement of additional factors. However, further detailed studies are needed to understand the mechanism and to identify the genes involved in the signalling pathways, which in turn could be responsible for inducing the observed apoptosis-like effect following infection. These interesting initial results suggest a possible correlation between the *M*. *agalactiae-* induced host cell damage *in vitro* and the tissue destruction observed during natural infections of small ruminants.

## Supporting Information

S1 Fig Immunofluorescence staining showing the early stages of chromatin condensation in *M*. *agalactiae*-infected HeLa cells. (PDF)

S2 Fig Western blot analysis of caspase-3 cleavage in *M*. *agalactiae-*infected HeLa cells. (PDF)
