# Supplementary material for: Mycoplasma agalactiae Induces Cytopathic Effects in Infected Cells Cultured In Vitro
Source: PLoS One. 2016 Sep 23;11(9):e0163603. doi: 10.1371/journal.pone.0163603 (PMC5035028; doi:10.1371/journal.pone.0163603)
Supplement: S1 Fig — (PDF) [file pone.0163603.s001.pdf]

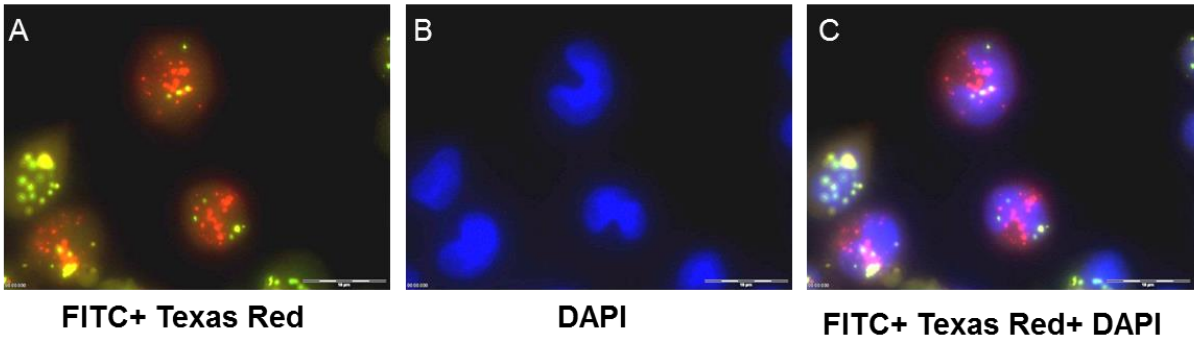

S1 Fig. Immunofluorescence staining showing the early stages of chromatin condensation in *M. agalactiae*-infected HeLa cells. (A) Double immunofluorescence staining with Texas Red and FITC revealing the invasion (red dots) and adhesion (green dots) of *M. agalactiae* at 30 h p.i. (B) DAPI staining of the same revealing the crescent shaped nuclei in infected HeLa cells. (C) Merged figure showing the invaded mycoplasmas over crescent shaped nuclei. Bars 10 µm.
