# Supplementary material for: Mycoplasma agalactiae Induces Cytopathic Effects in Infected Cells Cultured In Vitro
Source: PLoS One. 2016 Sep 23;11(9):e0163603. doi: 10.1371/journal.pone.0163603 (PMC5035028; doi:10.1371/journal.pone.0163603)
Supplement: S2 Fig — (PDF) [file pone.0163603.s002.pdf]

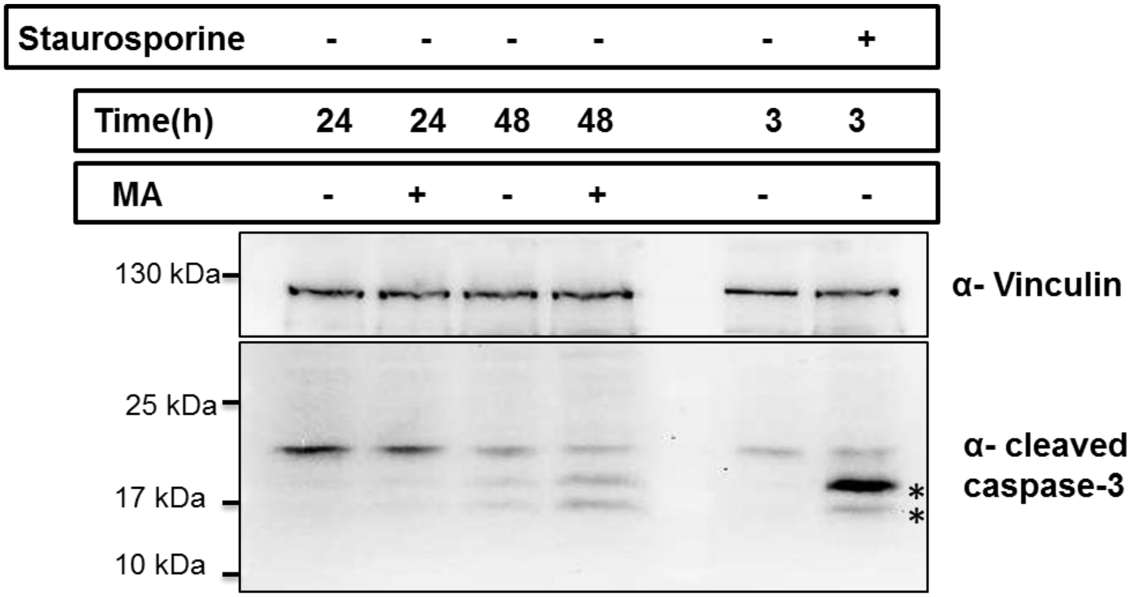


**S2 Fig. Western blot analysis of caspase-3 cleavage in *M. agalactiae* (MA) infected HeLa cells.** Both infected and uninfected cells were collected at 24 and 48 h p.i. and equal amount of cell lysates were blotted against caspase-3 antibody. Staurosporine served as positive control for cleaved caspase-3 and Vinculin was used as loading control. The cleaved products p19 and p17 are indicated by an asterisk (*).
